# You BETer be aware – learnings from a negative Phase 1 study

**DOI:** 10.18632/oncotarget.26898

**Published:** 2019-05-07

**Authors:** Sophie Postel-Vinay, Matthias Ocker

**Affiliations:** ^1^ Drug Development Department (DITEP), Gustave Roussy Cancer Campus, Paris-Saclay University, Villejuif, France; ^2^ NSERM, UMR981, ATIP­ Avenir Group,Villejuif, France; ^3^ Translational Medicine Oncology, Bayer AG, Berlin, Germany

**Keywords:** epigenetics-3, BET inhibitor, Phase 1, modelling, tox­

Anticancer drug development is aiming to identify distinct alterations in cancers that allow for a specific and potent inhibition of tumor cell growth and induction of cell death without affecting normal tissues. Besides genetic alterations like activating mutations in oncogenes (e.g. *EGFR* in lung cancer) or loss-of-function mutations in tumor suppressor genes (e.g. *BRCAJ /2* in breast and ovarian cancer), epigenetic mechanisms represent an attractive second layer of targets for oncology drug development.

The first wave of approved epigenetic anticancer drugs comprises DNA methyltransferase inhibitors (DNMTi) and histone deacetylase inhibitors (HDACi). These compounds are clinically used in hematologic malignancies and cutaneous T-cell lymphoma but are associated with high toxicity, poor selectivity and very little efficacy in solid tumors. To overcome those limitations, a second wave of epigenetic drugs targeting notably epigenetic writers, such as histone lysine methyltransferases, and epigenetic readers, like bromodomain and extraterminal motif (BET) family members, are currently in early phases of clinical development [1, 2].

We recently reported the results of a Phase 1 study with the novel and highly selective BET inhibitor BAY 1238097 in patients with advanced solid tumors [3]. BAY 1238097 showed promising efficacy in different preclinical lymphoma and solid tumor models [4–6]. Despite early signs of the expected pharmacodynamic potency of the compound in humans (e.g. on-target transcriptional suppression of MYC), the study had to be terminated prematurely due to the occurrence of unexpected non­ hematologic dose-limiting toxicities. Patients experienced various forms of pain (headache, back pain, myalgia) from the first dose level. Preclinical safety data did not indicate such risk - although pain is difficult to study in animals. Results from other BET inhibitor Phase 1 trials, in contrast, rather indicated hematopoietic and liver toxicity as well as nausea and fatigue as most common adverse events [1]. Also, our own experience from treating more than 240 patients with 15 different epigenetic drugs in Phase 1 trials rather pointed towards a late onset of toxicities [7], contrasting with the rapid appearance of pain symptoms as soon as the first dosing with the BET inhibitor BAY 1238097 [3].

The rapid onset of toxicities suggests a mechanism that may not be directly linked to BET inhibition. Indeed, BAY 1238097 was shown to bind to human adenosine transporter at an exposure that was reached at C_max_ in affected patients and could lead to increased concentration of purinergic pain mediators, which are strong inducers of pain and related symptoms [8]. In a back-translational approach, modelling was applied in the study to determine whether modifications of the dosing schedule could reduce those adverse events. Yet, this approach was not able to identify a regimen that would maintain exposure in the predicted efficacious range and reduce C_max_ below the level observed in patients experiencing DLTs. While binding to adenosine transporter may be considered an off-target effect, further effects directly linked to BET inhibition cannot be excluded. Transcriptional changes in BET target genes like *MYC* or HEXIM1 were observed already 30 min after dosing of BAY 1238097 in patients (see supplementary data in [3]) and it is not known if further mediators related to pain were also modified directly or indirectly e.g. via miRNA signaling. Toxicity profiles of other BET inhibitors currently evaluated in phase 1 trials will help answering this question.

BET proteins, like most epigenetic targets, are ubiquitously expressed and central regulators of gene activities. BET inhibitors may thus broadly alter gene transcription and the therapeutic window of these compounds may be narrow, unless robust biomarkers allow obtaining a descent therapeutic window. Excepted for NUT fusions in rare NUT midline carcinomas, such potential predictive biomarkers (including Myc amplification or BRD4 overexpression) have unfortunately not yet proven useful. Our study - like all phase 1 studies evaluating a BET inhibitor which have been reported so far- did not implement a patient selection biomarker in the dose-escalation phase.

Early phase drug development has a high risk of failure. Yet, not all negative studies are published although those results provide valuable information and are of importance to the field [9]. Our results show that BET inhibitors lead to an expected and rapid modulation of known target genes that can be used as pharmacodynamic biomarkers. These data, together with pharmacokinetic data and clinically observed adverse events can be used for modelling and simulation on how to overcome toxicity in an adaptive manner which helps to reduce trial costs and can shorten timelines. Although not all hypotheses on how BAY 1238097 induced the observed toxicity could be validated, several novel aspects were discussed that should be considered in future Phase 1 studies with such compounds, as we do consider BET proteins as a valid target for oncology drug development and, although unlikely, a class effect cannot be completely ruled out at this early stage where very limited results of other compounds have been disclosed.

Publishing negative trial results helps to optimize resources and financial efforts in drug development by preventing patients being exposed to inefficient or wrong drugs for their respective disease. In fact, clinical trial transparency and public disclosure of results has become mandatory for publication since more than 10 years by publishers (International Committee of Medical Journal Editors, ICMJE), by public and regulatory authorities such as WHO, FDA or EMA, and more recently also from funding public and private funding organizations [10]. Still, too many negative studies are underreported and subject to publication bias [11]. The disclosure of such data contributes to increasing trust and credibility of researchers, scientists and industry partners, which is important to emoll patients committed to trial participation in the future. In medicine like in science, a negative result is a result and we should learn from it, rather than ignore it. We therefore encourage all colleagues to share negative results for the best of drug development and patients in clinical trials.
